# The Contribution of Equitation Science to Minimising Horse-Related Risks to Humans

**DOI:** 10.3390/ani6030015

**Published:** 2016-02-23

**Authors:** Melissa Starling, Andrew McLean, Paul McGreevy

**Affiliations:** 1 Faculty of Veterinary Science, University of Sydney, Sydney NSW 2006, Australia; paul.mcgreevy@sydney.edu.au; 2 Australian Equine Behaviour Centre, Broadford VIC 3658, Australia; andrewmclean@esi-education.com

**Keywords:** horse-riding, ethology, safety, equitation science, learning theory

## Abstract

**Simple Summary:**

Equitation science describes an approach to horse training and riding that focuses on embracing the cognitive abilities of horses, their natural behaviour, and how human riders can use signalling and rewards to best effect. This approach is concerned with both horse welfare and rider safety, and this review discusses how equitation science can minimise risk to humans around horses and enhance horse welfare.

**Abstract:**

Equitation science is an evidence-based approach to horse training and riding that focuses on a thorough understanding of both equine ethology and learning theory. This combination leads to more effective horse training, but also plays a role in keeping horse riders and trainers safe around horses. Equitation science underpins ethical equitation, and recognises the limits of the horse’s cognitive and physical abilities. Equitation is an ancient practice that has benefited from a rich tradition that sees it flourishing in contemporary sporting pursuits. Despite its history, horse-riding is an activity for which neither horses nor humans evolved, and it brings with it significant risks to the safety of both species. This review outlines the reasons horses may behave in ways that endanger humans and how training choices can exacerbate this. It then discusses the recently introduced 10 Principles of Equitation Science and explains how following these principles can minimise horse-related risk to humans and enhance horse welfare.

## 1. Introduction

Equitation science is defined as the art and practice of horsemanship and horse-riding [1]. It is based on the founding principle of promoting the use of an evidence-based approach to explain and emphasise best practice in horse training and riding [2]. It is an inclusive system that aims to embrace all forms of training that are effective and ethical [3]. Equitation embraces a cost-benefit approach to ethics. The greater the impact to horses of a practice, the stronger the justification for that practice needs to be [4]. Ideally, all impacts could be considered moderate or less [4,5]. It also offers guidelines about what may be considered ethical. For example, positive punishment is where a noxious stimulus is used to suppress behaviour. This is discouraged in ethical equitation, as will be discussed later in this article. The uptake of ethical equitation has been broad and encouraging, but there has been some resistance for various reasons, such as the attitude of riders towards science, a preference for instruction from those successful at elite levels, and an emotional appreciation for finding harmony with horses organically [6]. Clearly, traditional approaches to riding instruction have been recorded over centuries in the teachings of the so-called ancient masters [7,8] as well as more contemporary horse trainers [7,9]. Many of these approaches, but not all, withstand scientific scrutiny [10].

Equitation science has a strong focus on horse ethology, acknowledging the way horses learn and their adaptive behavioural tendencies [11]. In this way, riders and trainers can be guided to work with horses in ways that are within the species’ cognitive limitations [12]. This is particularly important because horses and humans have different cognitive abilities [13]. Humans tend to attribute their own cognitive abilities to non-human animals [14,15,16], which can lead to unrealistic expectations about how quickly a horse can learn. The use of a concept such as “respect” may be important to the human but have little meaning for the horse. When training animals, trainers may become frustrated or assign human values and intentions to what are natural horse behaviours or cognitive limitations [17]. This in turn could lead to horse behaviour being misinterpreted as being deliberately challenging or wilfully disobedient, and the trainer adopting punishment to correct it [18]. Punishment is likely to impact negatively on equine welfare, but may also shift the focus of the horse to finding safety and relief from conflict rather than performing cued behaviours that are not relevant to their current goals [19]. This could lead to defensive behaviours or flight responses that threaten the safety of horse riders and trainers. Indeed, horse-riding is known to be a more dangerous activity than motorcycling, with most injuries occurring to the head, trunk, vertebrae, and wrists [20].

## 2. Causes of Dangerous Behaviour in Horses

### 2.1. Flight Responses

Like many animals, horses are often at their most dangerous both to themselves and to humans when highly aroused and attempting to escape perceived danger [21]. Horses are a large prey species reliant chiefly on flight at speed to keep themselves safe. This poses a problem for them in many human–horse interactions, as it is common for horses to be confined or restrained to varying degrees when around humans [22]. Being contained where the horse is worked with or housed reduces a horse’s options for gaining safety. If sufficiently aroused, it may run blindly into fences or other structures, or into humans [23]. More commonly, inability to escape may pressure the animal into experimenting with confrontational behaviours such as charging, striking with hooves, and biting [24]. These behaviours are likely to meet with some success, since humans confronted by a large, aggressive animal will naturally prioritise their own safety and attempt to escape by retreating [23]. The alternative is for the human to meet confrontation with escalated aggression and intimidate the horse into withdrawal. The consequences of these actions are potentially serious. In the former scenario, the horse’s aggressive behaviour is negatively reinforced by the human retreating, (*i.e.*, this has proved a successful way for it to create the space it needs to feel safer), or to avoid an event it had anticipated and found threatening or noxious [25]. Not only is this dangerous for the human, but it probably also perpetuates heightened arousal and negative emotional states in the horse [3]. Emotional conditioning may result in humans and/or training scenarios acting as signals for horses to become aroused and avoidant, or vigilant in anticipation of negative outcomes. Such anticipation primes animals to respond more strongly to aversive stimuli [26] and to interpret ambiguous signals as more likely to be potentially threatening [23]. This is unlikely to be comfortable for the horse, and is likely to trigger further defensive, high-arousal behaviours [12]. Furthermore, behaviours that have resulted in successful avoidance of an anticipated aversive experience can quickly become entrenched and very difficult to eliminate [27]. Deviating from avoidance behaviours known to be successful increases risk, so persistent avoidance behaviours are adaptive [28]. Re-training a horse that has learned undesirable avoidance or distance-increasing behaviours may prove challenging [29].

Punishing avoidance or distance-increasing behaviours is a strategy that comes with its own significant risks. If it is accepted that horses are at their most dangerous when trying to escape, then introducing further threats to a horse’s sense of safety in the form of pain, or eliciting flight responses with predatory behaviour, such as chasing, stalking, and sudden movements, could potentially contribute to the underlying problem rather than improving it [19]. While punishment may successfully suppress some dangerous behaviours, it may serve to heighten the horse’s arousal and negative emotional state while around humans, which could lead to the expression of other unsafe behaviours that have not been specifically punished [30]. Unwelcome behaviours may represent manifestations of the combined effects of arousal and emotional state.

### 2.2. Confusion and Conflict

A horse cannot necessarily read the intentions of its rider or trainer. There are many ways a rider or trainer’s behaviour can induce confusion. For example, signals may be unclear or inconsistent, or the horse may be unaware of how to reduce pressure imposed on it, or what the consequences may be [31]. This confusion can lead to a horse becoming conflicted, with opposing motivations, such as both approach and avoidance competing to manifest as its behavioural response. Such a horse may become uncertain of which behavioural response to adopt and, if arousal is elevated, this could prompt it to experiment with inappropriate locomotory responses such as bolting, bucking, rearing or shying [32]. Such loss of predictability and controllability may render the horse increasingly insecure, repeating the cycle of conflict and further endangering the rider.

### 2.3. Frustration

Aggression has often been shown to stem from frustration [33]. In applied ethology, the term frustration is used to describe thwarted motivation [34,35,36]. In horses, frustration may be caused by them being unable to perform natural behaviours due to restraint [37]. It is believed that frustration may be responsible for some stereotypies and self-injurious behaviours [38,39], but it may also negatively affect a horse’s behavioural inhibition and acceptance of handling procedures that might normally be tolerated [40]. Frustration can also arise during training in-hand and under-saddle, when reinforcement is inconsistent or non-existent and, as a result, horses are confused about how to escape pressure applied to them, or access other reinforcers, or avoid punishment [31]. This may have the effect of reducing self-regulation and increasing ambivalence so that horses may attempt to escape, becoming aggressive towards objects around them, including humans, or, alternatively, they may resort to apathy.

## 3. The 10 Principles of Ethical Equitation

Equitation science focuses on interacting with and managing horses in ways that avoid provoking dangerous behaviour in the first place [20]. This circumvents the aforementioned problems that can arise when addressing dangerous horse behaviours. Avoiding flight responses and minimising confusion and frustration in horses are the cornerstones of promoting safe behaviours and avoiding dangerous behaviours in equitation. Equitation science seeks to apply scientifically obtained data to training and riding horses to improve the safety and wellbeing of both horse and rider. It is not a single system or method, but it allows all methods of horse handling, training and riding to be assessed on the basis of a cost-benefit analysis that embraces their effectiveness and humaneness. There are significant challenges in empirically assessing the relative merits of approaches that are underpinned by the International Society for Equitation Science (ISES) principles because many other approaches to training include elements that align with these principles. If equitation does indeed guide horse riders and trainers towards safer interactions with horses, it is difficult to justify encouraging a deviation from this path simply to test its validity. Seeking horse riders who embrace either all of the principles of equitation science or a consistent subset of them to comparable degrees and skill in application remains problematic, so there are few data to support the effectiveness of the following ISES principles in isolation, (but any data that are available are cited). The cornerstones of promoting safety in equitation manifest in ISES’s recently released 10 Principles of Ethical [41]. They are as follows:

1. Train according to the horse’s ethology and cognition.
2. Use learning theory appropriately.
3. Train easy-to-discriminate signals.
4. Shape responses and movements.
5. Elicit responses one-at-a-time.
6. Train only one response per signal.
7. Form consistent habits.
8. Train persistence of responses (self-carriage).
9. Avoid and dissociate flight responses (because they resist extinction and trigger fear problems).
10. Demonstrate minimum levels of arousal sufficient for training (to ensure absence of conflict).

This article explains how these principles can minimise horse-related risks to humans as well as the welfare implications for horses.

### 3.1. Train According to the Horse’s Ethology and Cognition

***Ethology*** *is the study of animal behaviour in light of how a species has evolved to live*. A horse’s ethology informs what is known of horse social structures, including complex, dynamic social organisation with social rank determining which individuals receive priority access to resources. Equine ethology also shows that horses readily form attachment bonds and need the company of their own species, so isolation is detrimental [42]. A horse that is in an inappropriate social group may be less responsive during training and, equally, a horse that has encountered inconsistent training may be more likely to be distressed by marginally frustrating aspects of its world when not being ridden [3]. Horses have evolved to graze for about 16 h a day, which means they are moving for much of the day [29,43]. This has implications for horse management, as restricting physical movement ignores the motivation horses have for locomotion, and may result in frustration, post-inhibitory rebound [44] and subsequent behaviour problems [45].

***Cognition*** *refers to the ways animals process information about the world*. The equine prefrontal cortex is comparatively small to that of a human [46], so horses and humans may recall events differently from the way humans do [47]. Horses have evolved as prey animals that must be constantly aware of potential dangers, so they have developed excellent abilities to recognise stimuli that trigger responses such as the flight response [13]. Equine and human intelligence are qualitatively different, so care must be taken not to overestimate what they can perceive when it comes to which behaviours are “right” or “wrong” [12]. It is likely that incorrect behaviours are a product of training rather than a horse being wilfully disobedient. Equally, horses, like other animals, can develop emotional responses to stimuli that motivate their behavioural responses [48], so it is important not to underestimate their ability to react in a highly emotional way, which can make them unpredictable and dangerous, and have a significant impact on their welfare and willingness to work with humans [49,50].

Understanding equine ethology and cognition can help keep riders and trainers safe by helping them to appreciate what is most important to horses from moment to moment [51]. Understanding that horses must be vigilant and react very quickly to potential threats explains why they may be highly attentive to stimuli that signal threat, or even stimuli unfamiliar to them [29]. It is safer to assume that there is danger and act in the interests of self-protection than to assume that there is no danger and risk being injured or killed [52]. While a horse’s safety is dependent on speed, their agility is limited in small spaces by their size. This should inform those working with horses that, in the face of threat, horses will have an urge to run, and the more startled or frightened they are, the more powerful this urge will be [20]. They often run without any apparent regard for their own safety, seemingly when above certain arousal thresholds to be unable to notice or process how to navigate obstacles. If they are not so aroused as to flee in this way, they may be able to trial alternative behaviours to reduce the perceived threat. However, the more aroused they become, the more they will default to natural, energetically and pathologically costly behavioural solutions.

The ability to predict how horses may respond to threatening stimuli is a feature of what is known as horsemanship [53] and, as such, it arms riders and trainers with valuable information against provoking dangerous horse behaviour. Understanding ethology can also help predict the kinds of stimuli that may provoke extreme responses in horses. Horses are neophobic, so any unfamiliar stimuli may be perceived as potentially threatening, and young and/or inexperienced horses are likely to be triggered to escape by stimuli to which more-experienced horses may have habituated [20]. Furthermore, a horse that has been triggered to escape is likely to recognise the stimuli that started this response very well, and may also recall other contextual stimuli, and associate them with the flight response [31]. This could result in a horse being triggered to escape by stimuli to which it had been previously habituated. Addressing these strong and problematic associations takes patience and care to slowly build up a horse’s tolerance to stimuli that have previously set off a flight response [31].

Appreciating the social and locomotive needs of horses and being sure to meet them aids in avoiding frustration. Frustration can be a serious problem, as it can foster aggression [33].

### 3.2. Use Learning Theory Appropriately

Learning theory informs the ways horses learn that are common to all animals, and includes habituation, sensitisation, operant conditioning, shaping, and classical conditioning [25].

***Habituation*** *is recognised when animals stop responding to events and stimuli as they become accustomed to them*. As discussed above, horses are innately neophobic and often find characteristics such as the size, novelty, proximity and sudden appearance of stimuli frightening or startling [54]. Movement may mimic more threatening stimuli, such as stalking or rushing predators or conspecifics, particularly if it is sudden, erratic, or advancing towards the horse [3]. This may overcome even familiar stimuli and provoke unexpected responses. Habituation can reduce the intensity of reactions to aversive stimuli and be facilitated by desensitisation. For example, horses may be gradually exposed to an aversive stimulus with increasing intensity while ensuring that they remain in a calm state [55]. The horse learns that a calm response is more relevant than an aroused and fearful response.

***Sensitisation*** *is when an individual’s response intensity is increased*. In contrast to desensitisation, repeated exposure to arousing stimuli results in an increased likelihood of a response of increased speed or intensity to other stimuli as well. Being able to recognise this process when it is occurring is important when managing a horse during exposure to potentially threatening stimuli. It is worth noting that sensitisation to pressure cues from the rider is often desirable in equitation [56].

***Operant conditioning*** *describes training using rewarding or aversive consequences*. Such consequences are shown to be either reinforcing or punishing by their effect on the preceding behaviour. Therefore, if behaviour reduces in frequency, duration or intensity, the behaviour has been punished, whereas if it increases in frequency, duration or intensity, it has been reinforced. A behaviour can be punished by *applying* a noxious stimulus, which is known as positive punishment, or by *removing* a desired stimulus, which is known as negative punishment. Similarly, positive reinforcement describes the addition of a desired stimulus, and negative reinforcement the taking away of a noxious stimulus. It is important to understand which of the four operant processes is taking place. Punishments suppress behaviour, and may have suppressive effects not only on the behaviour that was punished, but also on contextual aspects and the horse’s future willingness or unwillingness to offer new behaviours [57]. In contrast, reinforcement encourages behaviours, particularly approach behaviours, when the reinforcement used is positive reinforcement [58]. Negative reinforcement is used extensively in horse-riding, with physical pressure being applied to parts of the horse’s body and being released when the desired behaviour is performed [57] (*i.e.*, the release of pressure is reinforcing). It is therefore imperative that the release of pressure be prompt, consistent, and easily achieved by the horse [3]. Negatively reinforced responses that rely on aversive stimuli (such as most rein or leg signals) should be continuously checked and maintained in order to avoid problem behaviours that may lead to reduced welfare [31] (*i.e.*, so that the horse does not habituate to the aversive stimulus).

***Shaping*** *is the gradual step-by-step building of behaviours, by reinforcing each step*. The targeted behavioural goal is achieved by rewarding successive approximations so that each step should differ only slightly from the previous step [57]. The advantage of this approach is that it enables horses to have many successes on the way to learning the final behaviour, which is likely to encourage positive emotional states associated with training [31].

***Classical conditioning*** *uses cues and signals to trigger and elicit behaviours*. They must be precisely timed to coincide with the start of the desired behaviour, and in that way, over time, they become cues that predict and trigger the desired behaviour [59].

It is critical to use learning theory appropriately and with skill. The implications of using it incorrectly are broad. The use of aversive stimuli in training to punish a behaviour are likely to negatively impact the horse’s mood [60]. Where an animal’s experiences are frequently aversive, it will expect more aversive experiences. This may make it more flighty and defensive. Furthermore, incorrect use of operant conditioning and errors in shaping can lead to frustration, which can, in turn, lead to aggression or conflict behaviours [31]. Understanding how habituation, sensitisation, and classical conditioning work and having the ability to correctly identify when these processes are taking place enables riders and trainers to avoid horses developing negative associations with training-related stimuli and encourages horses to build positive or neutral associations. Negative associations lead to negative moods [50], triggering potentially dangerous problems that have already been discussed, such as vigilance, hyper-reactivity, and conflict.

### 3.3. Train Easy-to-Discriminate Signals

Operant and classically conditioned signals should be unique and easily discriminated, particularly for signals that modulate behaviours in opposite directions [25]. Some examples are up/down gait transitions and faster/slower gait variations. Signals that are blurred or ambivalent may lead to horses becoming confused and distressed, particularly if the consequences of error are aversive, such as the application of punishment, increased pressure, or apparently inescapable pressure [61]. This could produce frustration and conflict with its associated dangers, but may also, paradoxically, lead to an incorrect response, particularly when training behaviours that are relatively novel. When both the correct and incorrect response have been reinforced, confusion may manifest as either a default to commonly practised behaviours or a reversion to stress-related behaviours, such as escape, aggression or apathy. Furthermore, similar signals for different responses may lead to the horse offering the opposite response to what was requested. For example, if the conditioned stimulus for moving the legs both “faster” and “slower” are similar (because the rider’s legs are involved in both responses), then an incorrect discrimination is likely to lead to the horse increasing or decreasing speed when asked to do the exact opposite. Such confusion can lead to dangers for humans and can have welfare implications for horses.

### 3.4. Shape Responses and Movements

Training should begin by reinforcing any rudimentary attempts at the target behaviour [62,63] that of course is completely unknown to the horse. Expecting the horse to extrapolate the correct response can lead to confusion and frustration, and subsequent compromised welfare [64]. As discussed above, this has implications for the safety of riders and trainers as it increases the likelihood of aggressive or escape behaviours, but also may lead to decreased reliability in responding to signals, which could put both horse and rider in the path of immediate danger, particularly if the horse does not stop or slow when signalled [5].

### 3.5. Elicit Responses One-at-a-Time

Cues or signals should be given individually, with a clear separation between them. This ensures that contradictory or conflicting signals are not given simultaneously, which can lead to inhibition of both signalled behaviours [65], as well as behaviours declining in strength and, potentially, signals being confused. The timing of signals should also be considered so that signals closely align with the behaviours they are cueing. For example, horses have four fundamental gaits. In walk, there are four beats, in trot, two beats, and in canter, three beats. The optimal time to elicit a response is when the leg is in swing phase because on the contrary stance phase, the limbs are preoccupied by mechanical constraints [66]. The implications for this are that there are four moments in walk swing phase in which to elicit an accelerating, decelerating or turning limb response, two moments in trot, and three in canter. This principle has the same implications for safety as shaping responses and using signals that are easy to discriminate between.

### 3.6. Train Only One Response per Signal

Each signal should elicit only a single response, so that it is clear to the horse which response is being signalled. In the dressage domain, rein stimuli are often used both to make the horse arch its neck, and for deceleration signals. In addition, rein cues signal the horse to turn, so tightening reins to bend the horse’s neck could also mean deceleration, or an ambiguous turn signal. Finally, the rider’s legs are frequently used for all the various locomotory effects as well as for turning and so called “bending” the horse’s body. There is also a strong potential here for confusion, and this use of one signal for several possible responses also violates the next principle of consistency.

Training only one response per signal is especially critical when separating acceleration and deceleration signals, and signals for speed and direction. Ambiguous signals lead to confusion in the horse, and a variety of broader effects on behaviour. Evidence in humans shows that ambiguity is avoided as aversive [67], and it can produce context-specific responses in animals [68] that may result in horses performing behaviours that are unexpected by the rider and may be punished, or produce behaviours from the human that are unexpected by the horse. This may in turn lead to increased conflict behaviours, incorrect behaviour at critical moments, hesitation, frustration, aggression, and ongoing anxiety surrounding being ridden [20], all of which may produce horse behaviours that endanger riders. One response may have multiple signals, but those signals should be exclusive to the one response and not be used to elicit other responses [31].

### 3.7. Form Consistent Habits

Consistency is a powerful tool. From a training perspective, consistency in signals and what they mean across different contexts also leads to consistency in the horse’s responses [25]. Clearly, this outcome is desirable, and enhances rider and trainer safety by promoting reliability in the horse’s readiness and ability to perform behaviours smoothly and without hesitation when signalled [69]. Consistency in the trainer’s approach to shaping so that successive steps always follow a similar pattern no matter what behaviour is being trained helps a horse to predict how training sessions will progress and the likely next steps that will be reinforced. This will reduce frustration, encourage positive affective states and promote persistence in shaping sessions so that the horse will not become frustrated or show a reduction in response rate when criteria change during shaping.

The same approaches that make consistency in and between training sessions beneficial may also promote safety outside training sessions. Consistency in the sequence of activities handlers and carers conduct around horses, the way they move, the way they vocalise, and when and how they interact with horses can allow these animals to predict the chain of events that are relevant to them [53]. This may be a double-edged sword, as it can produce sensitisation to stimuli in some circumstances, but where there are no stimuli producing powerful avoidance responses, it may have the opposite effect and habituate horses to everyday activities and routines [54]. Horses that can predict the immediately following events and know them to be safe are animals that will be more relaxed, less vigilant to potential negative stimuli, less prepared to take evasive or defensive actions or to attempt escape, and may be more exploratory and less fearful [23].

Inconsistent training and behaviour around horses can lead to the development of ambiguous signals that are difficult for horses to reliably interpret and respond to. This decreases their control of outcomes, leading to insecurity, which leads to diminished feelings of safety. This may compromise rider and trainer safety by creating horses that are skittish and unpredictable as they try to anticipate and adapt to surprising behaviour from humans.

### 3.8. Train Persistence of Responses (Self-Carriage)

Self-carriage refers to the maintenance of learned behaviours that should not need constant signalling or correction, but which the horse will continue performing until signalled otherwise [31,70]. If ongoing signals are required throughout the performance of a behaviour, it can have detrimental effects on signalling. It may lead to dull responses where signalling becomes meaningless background “noise” [59]. It may also lead to sensitivity and hyper-reactiveness to other stimuli. If a signal involves tactile pressure, and is not released when the horse begins to perform the correct behaviour, the horse does not know how to escape the pressure, and may experiment with undesired and dangerous behaviours, such as bucking [71]. Rider safety can be compromised in both scenarios. Equitation science encourages the use of sparse signalling as well as self-carriage of behaviours so that signalling does not become constant [3]. This is in the interests of the horse, as it does not put the animal in states of conflict, but it is also in the interests of the rider by avoiding hyper-reactivity or a drop in responsiveness.

### 3.9. Avoid and Dissociate Flight Responses (Because They Resist Extinction and Trigger Fear Problems)

Research has suggested that horses displaying a fear response (either flight or fight) feature prominently in horse-related injuries to humans [72]. Flight responses are related to seeking safety, and as such, have unique characteristics that have become adapted to keep horses safe and to find safety in the future. Flight responses are resistant to extinction, because threats to safety are serious and the potential cost of judging a stimulus safe when it is not could be injury or death [27]. In contrast, the cost of judging a stimulus unsafe when it is safe is likely to be less serious, such as missing opportunities for access to or learning about important resources (e.g., nutritious food, shelter, or mates). Flight responses are also prone to spontaneous recovery, even after alternative behaviours have been trained [27]. So, avoidance behaviours may continue even when the emotional need for avoidance is no longer driving the behaviour.

Flight responses are associated with a host of physiological and cognitive changes that are extremely effective at helping horses remove themselves from perceived danger and avoiding that danger in the future, but those same changes can be problematic in training and riding, as well as making horses unsafe [20]. High arousal and increased muscle tone make a horse very physically responsive to stimuli so they are primed to flight at the first hint of potential danger [73]. This is likely to give them the best chance of finding safety in the face of threats, but it is also likely to result in skittishness and unpredictable responses as they process stimuli through the filter of their state of high alertness. Furthermore, high arousal is damaging to the execution of problem-solving skills and concentration [50]. When an animal is in acute distress, such as that brought on by an urge to take flight, problem behaviours such as bolting, aggressive displays, distance-increasing behaviours, or apathy are likely to emerge [64]. These behaviours can be dangerous for horses and for humans working with or around them, and can also create instant negative associations with stimuli that the horses perceive when their need to escape becomes powerful [64]. The horse is also likely to become firmly entrenched in any behaviour that seemed to assist escape, which can mean that a horse learns to engage in dangerous behaviour to avoid perceived threats [71]. It may be that just the appearance of those perceived threats (e.g., someone approaching with a lead rope) can provoke avoidance behaviours that have been successful in the past even before there is any further indication of potential threat [43].

It is believed that when horses are often exposed to threatening stimuli, they can become chronically stressed [74] and that chronically elevated cortisol concentrations may result in health problems that reflect compromised immunity [75] but it can also come with a host of other potential problems that are likely to negatively impact performance and rider safety. These include learning and memory deficits, conflict behaviours becoming ritualised, redirected aggression, and long-term insecurity leading to problems such as separation-related distress, stereotypies, fear of conspecifics and heightened neophobia [76]. Chronically stressed horses may also develop a negative expectation bias, which can result in reduced interest in accessing reinforcers, and a subsequent cycle of negative emotional states perpetuated by negative expectation bias and avoidance and escape behaviours. This cascade may prevent adjustment to expectations and lead to risk aversion that discourages the explorations that might give them access to reinforcers that could improve mood [74].

Thus, chronic stress can result in a horse that may be volatile and unpredictable, may behave aggressively, and present extreme escape behaviours that can unseat and injure riders. Such horses may also have difficulties learning alternative behaviours due to their negative expectations and their preoccupation with performing behaviours that have successfully delivered escape and safety in the past. Furthermore, high glucocorticoid concentrations associated with chronic stress can affect cognition and memory, enhancing memory consolidation for avoidance learning, but hampering recall of other memories [77]. Equitation science demands avoidance of provoking flight responses, which, arguably, present the most dangerous aspects of horse behaviour. Horses attempting to take flight may fail to notice humans in their way, may be compelled to barge past them, or may view them as an obstacle to escape that heightens their distress or provokes human-directed aggression [20]. It is important to realise that horses in such states are often incapable of responding to signals in the way they have been trained, and are probably prioritising their own safety. This usually means increasing their distance from fear-inducing stimuli by whatever means are available. Where they are unable to do this with escape behaviours, they may attempt to do so through aggression [37]. Furthermore, their focus on imminent danger is likely to lead them to misinterpret, signals, stimuli, and human behaviours that they may be both familiar and comfortable with as threatening.

Attempts at training alternative behaviours without addressing the cause of the flight response may introduce further conflict or may cause it to be expressed in new ways. In addition, the horse may identify additional threats, such as humans stalking or chasing it, and this risks the horse making a single-trial association between its high arousal state and stimuli it has formerly been comfortable with, including humans, mounting blocks, or tack [58].

Flight responses in horses are clearly dangerous on several levels. Equitation science emphasises avoiding provoking them in the first place, but where this has occurred, it is important to realise that such horses have probably also associated their threatened safety with stimuli or events that occurred or were present at the time of the expression of these flight responses [3]. Dissociating those signals from flight responses may be difficult and time consuming, with many trials needed, but it must be achieved, and with minimal further threats to the horse’s perceived safety. This is why equitation science also recommends that the original source of a flight response and conflict be ascertained and addressed. For example, dysfunctions in negative reinforcement of deceleration responses require that these responses be retrained through the correct use of negative reinforcement or combined negative and positive reinforcement.

### 3.10. Demonstrate Minimum Levels of Arousal Sufficient for Training (To Ensure Absence of Conflict)

As discussed, arousal levels are associated with performance. The nature of the association depends on the task to be performed. If the task is complex, requiring assessment of options, problem-solving, precision, or self-control, the relationship can be described as curvilinear or an “inverted-U”, (*i.e.*, the shape of a graph with performance on the y-axis and arousal on the x-axis), with poor performance when arousal is low and the quality of the performance increasing until arousal is at a moderate level before declining as arousal peaks [50]. At low arousal, performance is poor because arousal is linked to interest and motivation, which are needed for the investment of energy into a related task. Performance increases with arousal to reach its maximum at moderate arousal, where the optimal level of arousal promotes interest in performing the task and speed in performance without the detrimental effects of high arousal. Increases in arousal beyond its optimal level lead to ever-poorer performance, while at the same time facilitating bursts of speed or strength. This can be explained by attributes of animals in high arousal that may impair performance, such as increased muscular tonus reducing the capacity for precise movements, and the sharpening of focus onto a single task or stimulus that may make animals less attentive and unable to respond to other stimuli [78]. This means that highly aroused horses may struggle to process signals not directly related to the salient stimuli, hampering problem-solving and the detection of and appropriate response to other external stimuli [3]. It also amplifies the veracity of the earlier mentioned Principle 5, that responses should be elicited singly. 

There are several theories of arousal [79,80], and many attempt to account for deviations in this inverted-U pattern. It is posited that where the required task is simple and highly relevant to the consequences of performing it, higher arousal will generally improve performance in a linear fashion and there will be no dramatic decline at higher levels [79]. For example, where a horse’s goal is to escape a noxious stimulus, increased arousal will increase heart rate and blood pressure, serving to increase speed. The horse’s focus will narrow to fixate on escape routes. Such horses are now in a good state to get to safety as quickly as possible, but if they are required to perform a complex task in order to do so, such as negotiating a maze, they will need a lower level of arousal to accomplish their goal quickly and efficiently.

High arousal states may improve performance for simple, energetically costly behaviours, but it is possible that they come with additional risks other than lack of precision and impaired problem-solving and decision-making skills. Arousal is a state of readiness, so it follows that at higher levels it may come with ever-increasing likelihoods of very active behaviours, and an ever-increasing dependency on them to solve any problems or threats that may be encountered. In a large prey animal such as a horse, this could make them more prone to dangerous behaviours related to flight or aggression, regardless of why they initially became aroused. It is possible that being in the presence of an attachment figure may help them to moderate arousal [81]. As such, equitation science encourages trainers to aim for promoting the minimum arousal level required to perform the target behaviour [82]. Not only will this support the horse’s ability to adapt to changing conditions appropriately and respond correctly even to unanticipated signals, but it will also reduce the likelihood of dangerous flight responses and aggressive behaviour in the case of conflict or when startled, and thus keep trainers and riders safer [20].

## 4. Conclusions

Horses can weigh more than 500 kg and are prone to strong flight responses. The danger they present when highly aroused and fixated on creating distance between themselves and perceived threats cannot be overstated. Those working with horses may inadvertently trigger flight responses, or be seen as a threat to be escaped from or driven away by horses, depending on environmental conditions such as available space or the presence of other threats. Humans can behave in ways that confuse, frustrate and frighten horses, or a combination of all three. Minimising the likelihood and strength of flight responses by managing a horse’s arousal and emotional state is one element of the ISES 10 Principles of Equitation Science that may serve to guide human behaviour around horses to promote human safety. These principles also address training goals that serve to minimise frustration and confusion in horses during horse-riding and husbandry with best practice use of learning theory and an understanding of equine ethology. These principles are likely to enhance human safety by promoting consistency and responsiveness in horses and avoiding conflict during the training of new behaviours and signalling of known behaviours.
